# The meaning of being conscious during surgery with local or regional anesthesia–A phenomenological hermeneutic study

**DOI:** 10.1016/j.ijnsa.2024.100224

**Published:** 2024-07-05

**Authors:** Marie Häggström, Kerstin Brodin

**Affiliations:** Mid Sweden University Department of Health Sciences, Sweden

**Keywords:** Intraoperative care, Lived experience, Meta-paradigm, Nursing, Phenomenological-hermeneutic method, Regional anesthesia, Surgery, Vulnerability

## Abstract

**Background:**

With increasing prevalence of surgery under local or regional anesthesia, which allows patients to remain conscious during the intraoperative phase, there is a growing need to comprehend the lived experiences associated with this practice.

**Objective:**

This study aimed to illuminate the lived experiences of individuals who remained conscious during surgical intervention under local or regional anesthesia.

**Design:**

A qualitative design was chosen.

**Setting(s):**

Participants in the present study were recruited from three surgical wards located in central Sweden using a purposive sampling strategy. The surgical disciplines included ear, gynecological, hernioplasty, orthopedic, and vessel surgeries.

**Participants:**

Fourteen narrative interviews were conducted with individuals who had undergone elective surgery while conscious**.**

**Methods:**

Verbatim transcribed text was analyzed using a phenomenological-hermeneutic method.

**Results:**

The lived experience of being conscious during surgery was marked by feelings of hope alongside a sense of losing one's identity and experiencing destabilization. Structural analysis revealed two themes. The first theme, 'being in the hands of others', encompassed subthemes such as 'entering an unfamiliar environment and procedure,' 'losing foothold and a sense of self-identity,' and 'enduring unexpected or anticipated discomfort.' The second theme, 'managing the inevitable for future health concerns,' involved subthemes such as 'pursuing self-acceptance of the situation,' 'entrusting the professionals while seeking signs of a smooth procedure,' and 'Enhancing own resilience through continuous support.

**Conclusions:**

Beyond the patient's physical well-being during surgery, the OR team should acknowledge the "person" component and focus on their emotional and social needs in this vulnerable situation. The four meta-paradigms of nursing—person, health, environment, and nursing—significantly influence the conscious patient's experience.

**Patient or Public Contribution:**

No patient or public contribution

**What is already known?**

• Anxiety prior to surgery is common, and during procedure, the patient's body is frequently exposed.
• Patients who remain conscious and awake during surgery may be affected differently and nursing care can significantly influence the patient's experience.

Alt-text: Unlabelled box

**What this paper adds**

• Being conscious and awake during surgery involves balancing between hoping for improved health and coping with a loss of own control in an unfamiliar and highly technological environment.
• While vulnerability presents numerous challenges during surgery, factors such as providing information, timely nursing interventions and showing genuine presence may support the person in managing the situation.

Alt-text: Unlabelled box

## 1 Introduction

Being awake and conscious during surgery has become increasingly common. In line with the development of surgical and anesthetic methods, surgery is often being performed under local or regional anesthesia (LA or RA). As a result, a growing number of patients experience surgery while remaining aware of their surroundings in the operating room (OR) (cf; Ramachandran et al., 2023). Patient engagement can improve the quality of care and inform policies (Bombard et al., 2018). Therefore, healthcare providers should actively listen to each patient's unique experiences and perspectives regarding the quality of care, including in the OR.

## 2 Background

Today, the number and complexity of surgical procedures have increased owing to advances in surgical, anesthetic, technological, and pharmacological techniques, along with changes in health services, which favor the recovery and stabilization of patients, with greater control of guarding and maintaining airway (Schindelar et al., 2022), and better control of pain, nausea, and vomiting. This has reduced hospital stays, the risk of infection, and costs (Kessler et al., 2015). A review by Fiani et al. (2021) showed that surgery with LA or RA had shorter operating time, less post-operative nausea, lower incidence of urinary retention and spinal headache, and shorter hospital stay than those performed under general anesthesia. Anxiety prior to surgery is common, and during procedure, the patient's body is frequently exposed (Blomberg et al., 2018). The anxiety a patient may feel before any surgical procedure has been described as an adverse state of apprehension secondary to worry about surgery, diseases, and the unknown (Schindelar et al., 2022).

Nursing is also about giving a voice to patients through design feedback and incorporating technological advancements into evolving nursing knowledge (Johnson and Carrington, 2023). Although nursing research investigating patient participation in the intraoperative period has increased in the past 20 years, further research on intraoperative nursing remains relevant considering ongoing medical developments.

Earlier research has indicated that patients who remain conscious and awake during surgery under RA or LA may be affected differently, and how nursing is performed can strongly influence the patient´s experience during the entire intraoperative phase (Arakelian et al., 2018). Further research is needed to develop the intraoperative experience of patient care during the perioperative period to facilitate the process for the individual (Gustafsson et al., 2007). This study contributes to the existing body of evidence on the experiences of fully conscious patients undergoing surgery, with a focus on the phenomena and subjective significance to the individual. Studying the lived experience of consciousness during surgery under local or regional anesthesia is an important area of research that sheds light on the human experience during medical procedures. By delving deep into the lived experience of this topic, healthcare providers can understand how to improve care and support for patients undergoing surgical procedures.

## 3 Aim

This study aimed to illuminate the lived experiences of individuals who remained conscious during surgical intervention under LA or RA.

## 4 Method

### 4.1 Theoretical perspective

This study's theoretical perspective is grounded in the nursing meta-paradigm, which encompasses people, the environment, nursing interventions, and health. Within this meta-paradigm, the "person" refers to the patient and the recipient of care. Beyond addressing the patient's physical well-being, the "person" component also focuses on their spiritual, emotional, and social needs. The nursing meta-paradigm embraces a comprehensive approach to healthcare by considering these interconnected aspects. These meta-paradigm concepts highlight the central issues of the discipline, form the cornerstone of nursing practice, and equip nurses with holistic and person-centered nursing skills (cf. Fawcett and Desanto-Madeya, 2013)

### 4.2 Design

To gain access to people's lived experiences and the essential meanings of being conscious during surgery, a qualitative design was chosen, which is preferable when gaining an understanding of meanings and human experiences (van Manen, 1997). A lived experience is one that we simply have, without concluding anything from it. It is more felt than known and can be understood when we explain it conceptually and narratively (Ricœur, 1976). The phenomenological-hermeneutical method was selected in line with the principles outlined by Lindseth and Norbergh (2004) to elucidate and comprehend the essence of life experiences and the phenomena of being conscious and awake during surgery. The chosen method is a way to get from the recognition that phenomena are important in certain contexts to a good understanding of the phenomena's meaning.

### 4.3 Setting, procedure, and participants

To gain an understanding of the person's lived experience (Flemming et al., 2003), data generation was performed through individual interviews with participants who had their own experience of being conscious during surgery. Participants in the present study were recruited from three surgical wards located in central Sweden using a purposive sampling strategy. The surgical disciplines included ear, gynecological, hernioplasty, orthopedic, and vessel surgeries. There was no previous relationship between researchers and participants prior the study. Nurses and nursing managers in the operating departments (i.e., the daycare ward and preoperative registration department) were informed and educated about the study. The nurses provided initial information about the study to patients who met the criteria. All information about the study was delivered in written and verbal formats, and included the study purpose, procedure, voluntary nature of participation, right to withdraw, and confidentiality of the data. The participants consented to being contacted by leaving their names and contact information in sealed envelopes. All patients that were contacted participated in the study, no one dropped out. The interviewer collected sealed envelopes regularly, and the participants were contacted in the manner they preferred. The handling of all information regarding the participants and data collection took place according to strictly confidential research ethics, and the envelope was stored in a locked safe to which only the researchers for the study had access. Upon contact, the researchers inquired whether the participants were still interested in participating and whether they remembered the intraoperative phase of their surgery. One of the participants heard about the study, contacted the researchers, and indicated their willingness to participate.

The inclusion criterion was being an adult (≥18 years) with experience of being conscious during elective surgery performed under regional anesthesia (RA) or local anesthesia (LA). Exclusion criteria included the inability to express oneself in Swedish, having had a caesarian section, or having undergone surgery to diagnose a malignancy. The included participants comprised 14 persons (four men and ten women) aged 49–75 years (mean age = 63 years) who underwent a variety of surgical procedures.

The determination of the sample size was guided by the principles outlined in a model by Malterud (2016), explicitly considering the concept of information power. Guided by the model, the sample size was based on the following criteria: a narrower aim rather than a broad one, the presence of a theoretical framework, rich data from the interview dialogues, and the chosen analysis method. These considerations collectively determined whether the study would attain sufficient information power with a smaller or larger number of participants. Based on the criterion that a sample holding more information can reduce the need for a larger participant group, we assessed that a sample size of approximately 12–15 participants would be sufficient to achieve the required information power.

### 4.4 Narrative interviews

Data collection was initiated during 2018–2019 and completed in 2022 conducted by female author KB (who was a PhD student at the time), supervised by Professor MH with several years of experiences of qualitative research and interviewing (also female). The second and first author discussed the quality and agreed to proceed with the interview guide, with narratives providing rich data from individual interviews (Brinkman, 2014). The interviews took place at the participants’ homes, the university, or at the participants’ workplace. Owing to the pandemic, the last two interviews were conducted via telephone. All the interviews were recorded and transcribed. The interviewees were encouraged to describe their lived experience of being conscious during surgery, and what it meant for them. All interviews commenced with an open-ended question: 'Could you please describe your experience of being awake or conscious during surgery?' The subsequent questions explored participants lived experiences from the beginning to the completion of the surgical procedure. During the interview, participants were asked probing questions such as "Tell me more" or "How did that make you feel?", encouraging them to elaborate on their responses and share personal stories using their own words (cf Polit & Beck, 2021). A pilot interview was conducted and discussed with both authors, and the rich data were judged to be of good quality. Throughout all interviews, participants freely shared their lived experiences. The interview durations ranged from 37 to 80 minutes, with an average duration of 55 minutes.

### 4.5 Data analysis

The analysis in our phenomenological-hermeneutical research focused on elucidating the meaning of a lifeworld phenomenon. Phenomenology attempts to uncover the concealed meaning of lived experiences, and hermeneutics interprets this meaning. Thus, they are interdependent with mutual belonging. This convergence of researching the meaning of life world phenomena, and of interpreting human expressions derives from the philosophy of Ricoeur (1976). The method aims to move from the explicit content of the text to its underlying meaning.

#### 4.5.1 Interpretation

The interpretation method used in this study moved in a spiral fashion through three distinct phases: preliminary naïve understanding, detailed structural analysis, and comprehensive interpretation (Lindseth and Norberg, 2004).

The first phase, naïve understanding, provided an initial understanding of the meaning of the text as a whole. The interview text was read several times in keeping with a phenomenological approach, as described by Lindseth and Norberg (2004), to obtain a sense of the material in its entirety and to initiate the approach for the structural analysis.

The second phase, structural analysis, was conducted to explain the text. The structural analysis was conducted through a systematic, step-by-step process, with the patients’ stories considered as a whole. The interpretation of the structural analysis focused on identifying and formulating themes. The whole text was read again sentence-by-sentence and then divided into meaning units in accordance with the aim of the study and from the perspectives obtained from the naïve understanding. A meaningful unit could be one or more sentences, or just a few words related to the same meaning, depending on the shift in content (Table 1). Each meaning unit was thoroughly condensed into everyday language and abstracted to give meaning to being awake and conscious during surgery. No software was used. It was then reflected on and related to other meaning units, depending on its essential meaning. The researchers reflected on the text in relation to naïve understanding, and subthemes were formulated and abstracted into themes (Lindseth and Norberg, 2022).

**Table 1:** Example of the analysis process.

Meaningful units	Condensed	Abstracted statement	Subthemes	Theme
Then they asked if I wanted to listen to music, and I said yes because I thought I would avoid hearing… it can be a little scary with the saw. But that wasn't possible because you lie on your side during a hip operation, so I couldn't wear headphones…just had to accept…	They asked if I wanted to listen to music, I said yes because I would avoid hearing the scary saw. But then it wasn't possible because I had to lie on the side during the and therefore couldn't wear the headphones… just had to accept	Wanted to listen to music to avoid the scary noise from the saw, but couldn't wear the headphones	Enduring unexpected or anticipated discomfort	Being in the hands of others

In the third phase, comprehensive understanding, the text was read as a whole again with the research question, naïve understanding, and findings from the structural analysis in mind. A critical interpretation, called comprehensive understanding, was written (Lindseth and Norberg, 2022). This analysis was performed with critical reflection on and awareness of pre-understanding (Lindseth and Norbergh, 2004). In this phase, we elucidated the deeper meanings within the participants’ world, as influenced by Ricœur's ideas (1976), to generate a holistic understanding of the text. According to Lindseth and Norberg (2004), pre-understanding is inescapable, yet we are only conscious of certain aspects. Critical reflection can enhance awareness; hence, we enclosed our opinions in parentheses to avoid presupposing the meaning of the phenomena for the individual (Lindseth and Norberg, 2022). The internal consistency of the interpretation and its relationship with competing interpretations were carefully examined. Both authors, who had different specialties, read the data. Author KB, drawing on prior experience as an OR nurse, and Author MH, with extensive experience in the intensive care setting, contributed diverse perspectives. This diversity was considered a strength as it ensured that the results accurately reflect the intraoperative reality based on written narratives.

### 4.6 Ethical approval

The study complied with ethical research principles in accordance with the Declaration of Helsinki, which promotes respect for all human beings and their health and rights while participating in research (World Medical Association, 2013). There were no pre-existing relationships between the researchers and interviewees before they agreed to participate in the study. Each participant provided informed consent for participation in this study. They were fully informed of their right to withdraw from the study at any point without the obligation to provide a reason. Furthermore, all the participants were guaranteed complete confidentiality. Patients who underwent surgical procedures for cancer were excluded from the study for ethical reasons. This study was approved by the Swedish Ethical Review Authority (D-nr 2016-159-31 M) and the heads of the involved departments.

## 5 Findings

The findings illuminate the lived experience of remaining conscious during surgery, based on narratives from the 14 participants described below, all of whom were born in Sweden (see Table 2).

**Table 2:** Overview of informants, current and pre-experienced anesthesia methods Gender (*M* = Male, *F* = Female)FRA = regional, *L*= Local, GA = general anesthesia), current surgery (*E* = ear surgery, *G* = gynecological surgery, HP = hernioplasty, *O* = orthopedic surgery VS = vessel surgery,) and data collection (*H* = patient´s home, *U*= area of university, WP= patient´s working place, *T* = Telephone).

Pat	Age/Gender	Surgery discipline e/g/o/vs	Anesthesia method r/l	Pre-experiences of anesthesia method LA/GA	Interviews length in minutes
1	63/M	O	R	none	37
2	60/F	O	R	1 GA	72
3	69/F	G	L	1 GA	34
4	75/F	G	L	1 GA	46
5	72/F	E	L	3 GA, 1 LA	80
6	49/M	O	R	None	41
7	72 /F	VS	L	None	42
8	57/F	O	R	1 GA, 1 LA	51
9	69/F	G	L	1 LA	37
10	74/F	G	L	2 GA	68
11	52/F	G	L	1 LA	49
12	50/M	O	R	1 LA, 2 GA, 1 RA	42
13	53/F	VS	L	1 GA	57
14	74/M	HP	L	None	44

### 5.1 Naïve understanding

The initial understanding from the narrative was that individuals who remained conscious during surgery struggled to navigate through the inevitable and endure their circumstances by attempting to accept them. As patients awaited the completion of the surgical procedure, being awake and conscious signified a state of vulnerability and dependence on others. The participants described entrusting their bodies to the care of others. They went between hoping for improved health and experiencing feelings of loss of control and vulnerability in an unfamiliar and highly technological environment. Their desire was to be recognized as unique individuals rather than merely “the next surgery in the OR”. The situation was challenging, primarily because of a perceived lack of control and a dearth of information.

### 5.2 Structural analysis

**Fig. 1:**
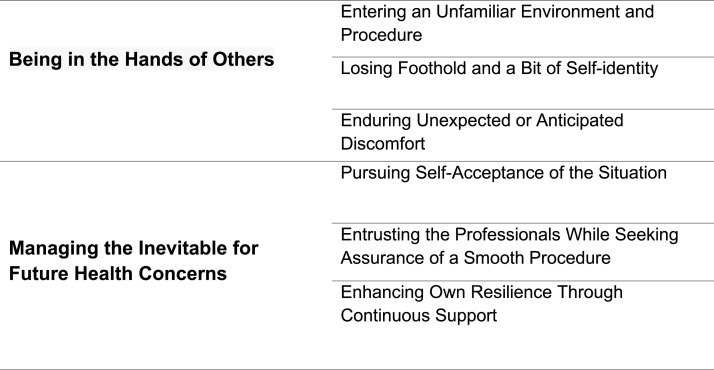
An overview over the findings.

The structural analysis revealed two themes with related subthemes: ‘*Being in the hands of others*’ and ‘*Managing the inevitable for future health concerns*’ (see Fig. 1).

### 5.3 Theme 1: being in the hands of others

The theme, 'Being in the hands of others', highlights the experience of being conscious during surgery and illuminates the vulnerabilities involved. Being in the hands of others in an unfamiliar environment awakens strong emotions, and obtaining information is essential. The theme is further elaborated on in the following subthemes: ‘*Entering an unfamiliar environment and procedure’,* ‘*Losing foothold and a bit of self-identity*’ and, ‘*Enduring unexpected or anticipated discomfort.*’

### 5.4 Entering an unfamiliar environment and procedure

The experience of consciousness during surgery was described as entering an unfamiliar environment and procedure. Upon arrival, the participants found themselves in the hands of the others. The intraoperative situation was perceived as somewhat daunting, with the OR environment characterized as ‘high-tech’ and ‘enigmatic.’ The atmosphere was cold and sterile and lacked an inviting quality. The presence of advanced medical technology in the OR prompted a range of emotions, including curiosity and admiration, among the participants. Participants valued being informed about the surgical procedure, as it helped them familiarize themselves with the unfamiliar. Information about surgery, whether provided or self-sought, triggered a mix of positive and negative emotions regarding the impending procedure. Some participants expressed a preference for a balanced level of detail, as exemplified by one participant: ‘*I knew how the procedure would go with an injection in the spinal cord and so on, so that I knew… although I didn't go on YouTube to watch how the actual surgery was performed because I felt it could be too scary. Because there was some breaking and sawing during the surgery. So, I didn't want to see that*’ (Interview # 3). The uncertainty of the operating room setting also elicited mixed feelings among the participants. They grappled with many issues, such as not being fully aware of the proceedings and not knowing the identities or numbers of individuals in the OR. One participant reflected, stating, ‘*Furthermore, it's interesting that there are people present during surgery, but I—the patient—isn't aware whether people are present or… whether it's good or bad… I don't know if it makes any difference or…*’ (Interview # 10).

### 5.5 Losing foothold and a bit of self-identity

The participants expressed a sense of dependency on others and an inability to cope independently during the surgical process. Feeling vulnerable in the unknown involved a loss of footing and partial loss of self-identity. Despite various treatments, nursing interventions, routines, and rules, participants acknowledged their complete dependence and reliance on others throughout the intraoperative period. This vulnerability in the unknown transformed the participants into a version of themselves with entirely different needs and concerns from the usual. One participant shared, ‘*I was surprised; normally I see myself as a person with confidence, but in the OR, I was lost, and I almost became someone else*’ (Interview # 9). This ‘other person’ they became in the unfamiliar situation was perceived as helpless and weak, yet they recognized it as a natural part of the circumstance.

Despite viewing this dependence as a natural aspect of the situation, participants were aware that their future health relied on the success of the operation and felt a heightened sense of vulnerability. Recognizing their limited ability to influence the surgical procedure increased their feeling of vulnerability. Uncomfortable with this dependence, some participants attempted to regain a semblance of control over the situation: ‘*I am not used to depending on others; no, I am used to having control in my life. In the OR, I could not really affect anything, so I think I tried to take some kind of control by just lying very still and hoping the surgeon had a good day*’ (Interview # 11). Maintaining a sense of identity and personality as much as possible in the operating room environment was crucial for the participants to accept the situation.

### 5.6 Enduring unexpected or anticipated discomfort

The participants frequently expressed discomfort associated with surrendering control and having limited knowledge or relevant experience during the procedure. Being in the hands of others meant enduring unexpected or anticipated discomfort, which explained the participants’ varying degrees of discomfort during surgery. Intraoperative routines required participants to perform specific actions, such as gaping, sticking out their tongues, remaining ‘absolutely still,’ or assuming different positions. In this unfamiliar and uncontrollable environment, participants felt compelled to obey, as expressed by one participant: ‘*…just do as you are told, and for your own safety, you do as you are asked to do*’ (Interview # 3). Some of these actions, while potentially exposing the participants, took meaning when explained with adequate information. For instance, one source of unexpected discomfort was the painful administration of anesthesia. Participants described experiencing unexpected pain and discomfort during the anesthesia procedure, which they deemed necessary but were unprepared for: ‘*It hurt a lot—the needle. It was the worst part of the whole thing, getting anesthesia*’ (Interview # 14).

Another source of discomfort was enduring and tolerating sensations during surgery. Noise from the saw during hip surgery caused discomfort. Feeling these sensations made the participants skeptical about the effectiveness of the anesthesia, heightening their discomfort.

‘*Then they asked if I wanted to listen to music, and I said yes because I thought I would avoid hearing… it can be a little scary with the saw. But that wasn't possible because you lie on your side during a hip operation, so I couldn't wear headphones…*’*/(*Interview # 14).

*It* was uncomfortable for them to experience their bodies being manipulated in ways that caused varying degrees of discomfort, mainly because of inadequate anesthesia. Some participants reported feeling pain during the surgical intervention, with more severe pain during ongoing surgery being the most vividly remembered intraoperative experience: “*…surgery with local anesthesia was a very unpleasant experience because it hurt terribly at times, but they said it was normal for this kind of surgery. I will never do it again*” (Interview # 6).

### 5.7 Theme 2: managing the inevitable for future health concerns

The theme 'Managing the inevitable for future health concerns' illuminate's participants' hopes and aspirations for improved health, as well as their efforts to endure and navigate the situation. Holding onto positive feelings such as trust made their efforts easier and the situation more manageable. The theme was further elaborated in the following subthemes: ‘*Pursuing self-acceptance of the situation’,* ‘*Entrusting the professionals but seeking signs of a smooth procedure’* and ‘*Enhancing own resilience through continuous support.*’

### 5.8 Pursuing self-acceptance of the situation

Hoping for a better life after surgery, participants viewed the procedure as a necessary step toward achieving improved or optimal future health. They described engaging in internal negotiations to navigate their acceptance of the intraoperative situation and actively pursuing self-acceptance. Despite feeling safe, a constant undercurrent of anxiety and nervousness persisted. Participants further expressed a lack of knowledge about what to expect and felt the need to ‘go with the flow’ and ‘do what they were asked to do’ to the best of their ability. Accepting the situation was challenging; however, a strong desire for improved health and well-being served as a motivating force.

Additionally, the participants grappled with the idea of being awake during the procedure and the uncertainty surrounding the experience. They reminded themselves of the advantages of being awake during surgery and acknowledged that the risks were lower than if they were under general anesthesia. The participants also felt great relief after the completion of the surgery and expressed profound gratitude.

### 5.9 Entrusting the professionals while seeking assurance of a smooth procedure

The operating room staff's behavior played a significant role in shaping the intraoperative experience and influencing how patients perceived safety. Entrusting the professional was essential; participants actively sought signs of a smooth procedure and closely observed staff actions. They noticed that if the staff seemed skillful and confident, they sought reassurance for a smooth procedure. When this was the case, participants felt safer, as illustrated by one participant: ‘*I observed them and… they were so professional, and everybody seemed to know exactly what to do*’ (Interview # 4). Additionally, participants found reassurance in the monitoring they received, such as monitoring heart rate (ECG) and saturation measurements. This monitoring provided a sense of security because it indicated that the staff had complete control. Because participants did not feel in control of the situation, they found it essential to feel comfortable relinquishing control over professionals.

### 5.10 Enhancing own resilience through continuous support

According to the participants, resilience during the intraoperative process was enhanced when they received continuous support throughout the procedure. Their resilience depended on whether the nurses in the operating room paid attention to and showed genuine concern for their well-being. One participant said, ‘*They kept contact with me, informed me of about the operation so I could follow the surgery steps and everything else important during the stay in the OR, that felt very secure*’ (Interview # 13). Having someone nearby during the procedure was expressed as an essential source of support. Whether it was having a hand to hold, a person to talk to, or someone inquiring about their well-being, this constant presence was deeply valued: ‘*This nurse beside me was solely focused on my care; she held my hand and asked if I felt alright… they were the most important person at that moment*’ (Interview # 7).

Supportive actions included receiving tailored information about the procedure step-by-step and being treated as an individual: ‘*If you wanted to engage in small talk, you got it; if you wanted to joke, it was allowed as well. In moments when I just wanted to relax and get through the surgical intervention, the staff showed respect for that*’ (Interview # 1). Supportive and symptom-relieving measures included medication and drug use. For instance, participants highlighted that when they had trouble relaxing or when the surgery took an extended time, they were provided with sedatives and calming drugs to endure the procedure. If the participants experienced pain during surgery, they appreciated that the nurse paid attention and provided support and relief through medication; knowing that medication was available if needed was important.

### 5.11 Comprehensive understanding and reflections

The study aimed to shed light on the lived experiences of consciousness during a surgical intervention conducted under local or regional anesthesia (LA or RA). Remaining conscious during surgery involves confronting personal challenges, resulting in a diminished sense of direction, identity, and stability. The overarching findings uncovered a vulnerability in the face of uncertainty, leading to feelings of disorientation and a loss of personal identity. This experience includes clinging to the hope of improved health to manage the stress and encompasses enduring both psychological and physiological distress as individuals entrust their bodies to others.

Participants in the study negotiated with themselves to endure unexpected discomfort, encompassing the pain caused by the procedure, surgical sensations, and bodily manipulations. They endured psychological and physiological distress, shedding light on several factors contributing to their ability to accept surgery while conscious. Striving to make the unavoidable manageable as a means to reach acceptance emerged as a coping strategy amidst the facilitators and challenges present. Furthermore, gaining acceptance was seen as a strategy to alleviate anxiety and enhance well-being, consistent with a study that explored the effects of acceptance and commitment therapy (Cao et al., 2022).

The findings will be further reflected in the context of the nursing meta-paradigm: *person, health, environment, and nursing (*Fawcett and Desanto-Madeya, 2013). Each meta-paradigm plays a key role in the nursing process and seems essential for providing care to conscious patients during surgery. The meta-paradigm of *person* focuses on the recipient of care. Individuals receiving care desire to be seen and acknowledged during the procedure but need to cope with a lot on their own. Maintaining identity and personality as much as possible in an OR environment helps individuals accept situations. Feeling vulnerable in the unknown means losing oneself to some extent, being forced to leave the body in the hands of others, trusting others and their knowledge, and yielding to a feeling of dependence. During the intraoperative period, a conscious person receiving care seeks to exert some form of control, with control being more of a feeling than a concrete fact.

The meta-paradigm of *health* refers to patient quality and wellness. Participants strived to accept and negotiate with themselves that undergoing surgery was vital for regaining their health and well-being, which is the wish and private goal of enduring surgery. Being in safe hands and receiving supportive action can be understood as facilitators of acceptance when performed seriously and respectfully. Vulnerability related to the intraoperative experience means that the staff's consideration and professionalism in the care encounter are significant for the participants’ well-being during surgery, as suggested by Bergman et al. (2012). Professionalism involves maintaining a calm and reassuring atmosphere in the room so that the patient could feel at ease knowing everything was running smoothly. It also involves delivering information during surgery, which is essential for handling situations and unexpected sensations. These findings align with a study in which older people's experience of the perioperative period when undergoing hip or knee replacement was studied (Gustafsson et al., 2007). The participants in this 16-year-old study (which was similar to ours with updated data) were not fully informed and prepared for changes throughout the perioperative period. Insecurity, fear, and anxiety increase in patients when care and nursing measures are perceived as impersonal, and patients who experience being seen as objects more often than humans experience care as insensitive and distressing (Wassenaar et al., 2014).

The *environmental* meta-paradigm evokes a mixture of feelings, encompassing both insecurity and admiration for the conscious individual. A well-prepared and organized OR environment holds the potential to foster trust in patients and cultivate a sense of security. This finding is supported by Berg et al. (2013). The technology present in the OR was a contributing factor to the perception of being in a modern and secure setting. This finding aligns with similar results from other studies that have highlighted the role of technology in enhancing the sense of safety, particularly when it is deemed essential for healthcare delivery. These studies also emphasize that a contemporary and safe OR necessitates advanced technology (cf. Bayramzadeh and Aghaei, 2021).

The meta-paradigm of *nursing* refers to how nurses apply their knowledge and skills when caring for patients. It also refers to the attributes of the nurses who provide care. The findings showed that nursing interventions, especially timely interventions, are essential for conscious patients during surgery. Our findings regarding unexpected discomfort during conscious surgery are strengthened by a quantitative study conducted by Mitchel et al. (2008). Participants in their study, as in our study, were anxious about possibly feeling the surgeon's touch, seeing their body cut open, or surgery being more painful than expected, and vulnerability was associated with the provision of anesthetic information. Another study showed that patients experienced a high level of anxiety at the induction of anesthesia (Haugen et al., 2009) which was echoed in a study ten years later (Henningsen et al., 2018). These findings are comparable to the results of the present study, as participants described the intervention as painful and associated it with enduring discomfort. Discomfort also ensued from not fully trusting the anesthesia form given, mostly because of still being able to feel that they were being touched, as reported by the participants. Such unexpected discomfort has also been described in other studies (Bager et al., 2015; Henningsen et al., 2018). The intraoperative situation places individuals under immense physical and mental pressure (cf. Sigdel, 2015), and supportive nursing interventions are facilitators. Thus, nurses must consider patents’ vulnerabilities and guide them through the intraoperative process, which was also concluded by Ingvarsdottir and Halldorsdottir (2018). Individually tailored intraoperative nursing interventions are needed to make patients feel safe and relaxed in the OR (Arakelian et al., 2017; Kaptain et al., 2019; Yilmaz et al., 2020; Özşaker and Yeşilyaprak, 2018) which aligns with this study´s findings.

The overall findings highlight the challenges and facilitators when patients are conscious during surgery. Being conscious during surgery entails accepting a role in a complex, sensitive, and dependent situation, and temporarily losing a bit of one's identity. Understanding the emotional challenges associated with perceived loss of control can guide interventions and practices to improve surgical experience and foster holistic patient well-being. Various factors can facilitate or pose challenges in this vulnerable situation. How patients handle an intraoperative situation may depend on various factors, such as previous experiences and personality (Bergman et al., 2012; Karlsson et al., 2012), which stresses the importance of person-centered care in the OR. Enabling a patient to be involved in their own care is therefore important, which is evident in the findings of this study, as well as in those of other studies (Mauleon et al., 2007; Willassen et al., 2015). Therefore, it is important to promote individualized intraoperative care to the greatest extent possible, in which each patient's unique circumstances are considered in a quality care encounter.

The results of our study may not surprise an experienced nurse, but this does not make them any less important. Listening to patients' voices is crucial in healthcare; the stories they share provide valuable insights we might otherwise only guess at. By researching their lived experiences, we can ensure that care is patient-centered and that we address their actual needs rather than the needs we assume they have.

### 5.12 Limitations

The study acknowledges its limitations, primarily due to the inclusion of participants from only three surgical units in Sweden. Of these participants, only four were men, and all were born in Sweden, potentially influencing the results. Additionally, patients who underwent surgery to diagnose malignancy or cesarean section while awake were excluded, which also poses a limitation.

### 5.13 Trustworthiness

The description provided by Lincoln and Guba (1985) was utilized to discuss trustworthiness of this study. The results of phenomenological-hermeneutical research are not generalizations; however, the findings may be interesting and recognizable for staff at other hospitals (Lindseth and Norberg, 2022).

The *transferability of our findings* is reasonable, meaning that the results are relevant to similar contexts and situations. *Additionally, our* findings are applicable to different contexts, such as dental practices.To ensure *credibility*, each step of the analysis process was characterized by reflexivity and verification of the original text, which was then discussed with the research team. Any discrepancies were examined until a consensus was reached, and quotes were added. *Dependability,* which demonstrates stability over time and across conditions, ensuring that the findings are consistent and replicable, was achieved through a thorough description of the research process that was logical and well-documented. Additionally, the same research question was asked to all participants. Finally, *confirmability* refers to the degree of neutrality or the extent to which the findings of a study are influenced by respondents rather than researcher bias, motivation, or interest. The authors maintained a reflexive stance regarding the findings in terms of pre-understanding and existing theories. This meant that the authors were vigilant against their own biases, assumptions, beliefs, and preconceptions brought to the study, while also being aware that complete reduction of pre-understanding is not possible. Reflexive notes were written after each interview and continuously discussed, reviewed, and reflected upon (cf. Cypress, 2017).

### 5.14 Recommendation for practice

Ensuring high-quality care encounters by providing perioperative information tailored to the specific procedure and individual needs seems crucial. Comprehensive perioperative information that outlines the various sensations patients may experience and details about the surgical environment might help the conscious patient cope better. The OR team should be attentive to signs of discomfort, as many reports involve unexpected sensations such as pain, noise, and smell. Beyond addressing the patient's physical well-being during surgery, the OR team should acknowledge the whole "person" component and consider their emotional and social needs in this vulnerable situation. Acting and conversing professionally is important, as conscious patients often observe the room and the staff. Their anxiety might partly depend on how "normally" the team behaves and if there are any indications that something might go wrong.

## 6 Conclusion

The findings of this study provide valuable insights into the meaning of being conscious during surgery under local or regional anesthesia, offering opportunities to enhance the quality of care. It is not surprising, but worth noting, that the technical aspects of surgery itself may not be the only essential factors influencing how patients experience consciousness during surgery. The four meta-paradigms of nursing–person, health, environment, and nursing–all seems to have an impact on the experience of being conscious during surgery.

Being completely exposed as a person and body can trigger feelings of discomfort and anxiety. Although vulnerability presents many challenges during surgery, factors such as providing information about the procedure and environment, timely nursing interventions, and showing genuine presence can help the person manage the situation. Navigating the intraoperative scenario seems to be influenced by the OR team's ability to identify needs, offer perioperative information, and provide reassurance about the procedure.

## Fundings

No externa fundings.

## Declaration of generative AI and AI-assisted technologies in the writing process

During the preparation of this work the authors used ChatGPT in order to improve language. After using this tool, the authors reviewed and edited the content as needed and takes full responsibility for the content of the publication.

## CRediT authorship contribution statement

**Marie Häggström:** Writing – review & editing, Writing – original draft, Visualization, Validation, Supervision, Software, Project administration, Methodology, Investigation, Formal analysis, Conceptualization. **Kerstin Brodin:** Writing – original draft, Project administration, Methodology, Investigation, Formal analysis, Conceptualization.

## Declaration of competing interest

The authors declare that they have no known competing financial interests or personal relationships that could have appeared to influence the work reported in this paper.

## References

[bib0001] Arakelian E., Laurssen E., Öster C. (2018). Older patients' worries in connection with general anesthesia and surgery—a qualitative study. J. Perianesth. Nurs..

[bib0002] Arakelian E., Swenne C.L., Lindberg S., Rudolfsson G., von Vogelsang A.C. (2017). The meaning of person-centred care in the perioperative nursing context from the patient's perspective–an integrative review. J. Clin. Nurs..

[bib0004] Cypress B.S. (2017). Rigor or reliability and validity in qualitative research: perspectives, strategies, reconceptualization, and recommendations. Dimens. Crit. Care Nurs..

[bib0005] Bager L., Konradsen H., Dreyer P.S. (2015). The patient's experience of temporary paralysis from spinal anaesthesia, a part of total knee replacement. J. Clin. Nurs..

[bib0006] Bayramzadeh S., Aghaei P. (2021). Technology integration in complex healthcare environments: a systematic literature review. Appl. Ergon..

[bib0007] Berg K., Kaspersen R., Unby C., Frisman G.H. (2013). The interaction between the patient and nurse anesthetist immediately before elective coronary artery bypass surgery. J. Perianesth. Nurs..

[bib0008] Bergman M., Stenudd M., Engström Å. (2012). The experience of being awake during orthopaedic surgery under regional anaesthesia. Int. J. Orthop. Trauma Nurs..

[bib0009] Blomberg A.C., Bisholt B., Lindwall L. (2018). Responsibility for patient care in perioperative practice. Nurs. Open..

[bib0010] Bombard Y., Baker G.R., Orlando E. (2018). Engaging patients to improve quality of care: a systematic review. Implement. Sci..

[bib0011] Brinkmann S. (2014). Unstructured and semi-structured interviewing. The Oxford Handbook of Qualitative Research.

[bib0012] Cao J., Sun P., Zhang L., Chen X., Gui W., Ou A., Chen K., Ma L. (2022). Effects of acceptance and commitment therapy on self-management skills and psychological resilience of young and middle-aged patients underwent percutaneous transluminal coronary intervention for primary myocardial infarction: a pilot study. Trials.

[bib0013] Fawcett J., Desanto-Madeya S. (2013).

[bib0014] Fiani B., Reardon T., Selvage J., Dahan A., El-Farra M.H., Endres P., Taka T., Suliman Y., Rose A. (2021). Awake spine surgery: an eye-opening movement. Surg. Neurol. Int..

[bib0015] Fleming V., Gaidys U., Robb Y. (2003). Hermeneutic research in nursing: developing a Gadamerian-based research method. Nurs. Inq..

[bib0016] Gustafsson B.Å., Ponzer S., Heikkilä K., Ekman S. (2007). The lived body and the perioperative period in replacement surgery: older people's experiences. J. Adv. Nurs..

[bib0017] Haugen A.S., Eide G.E., Olsen M.V., Haukeland B., Remme Å.R., Wahl A.K. (2009). Anxiety in the operating theatre: a study of frequency and environmental impact in patients having local, plexus or regional anaesthesia. J. Clin. Nurs..

[bib0018] Henningsen M., Sort R., Møller A., Herling S. (2018). Peripheral nerve block in ankle fracture surgery: a qualitative study of patients’ experiences. Anaesthesia.

[bib0019] Ingvarsdottir E., Halldorsdottir S. (2018). Enhancing patient safety in the operating theatre: from the perspective of experienced operating theatre nurses. Scand. J. Caring Sci..

[bib0020] Johnson E., Carrington J.M. (2023). Revisiting the nursing metaparadigm: acknowledging technology as foundational to progressing nursing knowledge. Nurs. Inq..

[bib0021] Kaptain K., Ulsøe M.L., Dreyer P. (2019). Surgical perioperative pathways—Patient experiences of unmet needs show that a person-centred approach is needed. J. Clin. Nurs..

[bib0022] Karlsson A.-C., Ekebergh M., Mauléon A.L., Österberg S.A. (2012). Is that my leg?” Patients’ experiences of being awake during regional anesthesia and surgery. J. Perianesth. Nurs..

[bib0023] Kessler J., Marhofer P., Hopkins P.M., Hollmann M.W. (2015). Peripheral regional anaesthesia and outcome: lessons learned from the last 10 years. BJA: Br. J. Anaesth..

[bib0024] Lincoln Y.S., Guba E.G. (1985).

[bib0025] Lindseth A., Norberg A. (2004). A phenomenological hermeneutical method for researching lived experience. Scand. J. Caring Sci..

[bib0026] Lindseth A., Norberg A. (2022). Elucidating the meaning of life world phenomena. A phenomenological hermeneutical method for researching lived experience. Scand. J. Caring Sci..

[bib0027] Malterud K., Siersma V.D., Guassora A.D. (2016). Sample Size in Qualitative Interview Studies: guided by Information Power. Qual. Health Res..

[bib0028] Mauleon A.L., Palo-Bengtsson L., Ekman S.L. (2007). Patients experiencing local anaesthesia and hip surgery. J. Clin. Nurs..

[bib0029] Mitchell M. (2008). Conscious surgery: influence of the environment on patient anxiety. J. Adv. Nurs..

[bib0038] Özşaker E., Yeşilyaprak T. (2018). The problems of patients with stoma and its effects on daily living activities. Med. Sci. Discov..

[bib0030] Ramachandran G., Sundar A.S., Venugopal V., Shah H.D., Dogra N. (2023). Recent advances in cardiac anaesthesia. Indian J. Anaesth..

[bib0031] Ricoeur P. (1976).

[bib0032] Schindelar L., Townsend C.B., Ilyas A.M., Matzon J.L. (2022). The impact of intraoperative nursing care on perioperative complications during wide-awake local anesthesia hand surgery. J. Hand. Surg. Glob. Online.

[bib0033] Sigdel S. (2015). Perioperative anxiety: a short review. Glob. Anesth. Perioper. Med..

[bib0034] van Manen M. (1997).

[bib0035] Wassenaar A., Schouten J., Schoonhoven L. (2014). Factors promoting intensive care patients’ perception of feeling safe: a systematic review. Int. J. Nurs. Stud..

[bib0036] Willassen E., Blomberg A.-C., von Post I., Lindwall L. (2015). Student nurses’ experiences of undignified caring in perioperative practice–Part II. Nurs. Ethics.

[bib0037] World Medical Association (2013). World Medical Association of Helsinki: ethical principles for medical research involving human subjects. JAMA.

